# Supplementary material for: Direct observation of selective autophagy induction in cells and tissues by self-assembled chiral nanodevice
Source: Nat Commun. 2018 Oct 29;9:4494. doi: 10.1038/s41467-018-06946-z (PMC6206072; doi:10.1038/s41467-018-06946-z)
Supplement: Supplementary file 1 — Supplementary Information [file 41467_2018_6946_MOESM1_ESM.pdf]

Supplementary information

Direct observation of selective autophagy induction in cells and tissues by self-assembled chiral nanodevice

Sun et al.

Supplementary Note 1

All glassware was cleaned with freshly prepared aqua regia and rinsed thoroughly with deionized (DI) H_2O before use. All the reagents used in this study were purchased from Sigma-Aldrich (St. Louis, MO, USA). The DI water used throughout the study was obtained from a Milli-Q device (18.2 MΩ; Millipore). The MCF-7, HeLa, and 4T1 cell lines were obtained from the China Center for Type Culture Collection (Wuhan, P.R. China). The thiolated DNA oligonucleotides, purified with high-performance liquid chromatography (purity > 95%), were manufactured by Shanghai Sangon Biological Engineering Technology & Services Co. Ltd, and then suspended in TE buffer (Shanghai Sangon) at a final concentration of 100 μM. TEM images were obtained with a JEOL JEM-2100 microscope at an acceleration voltage of 200 kV. The particle size distributions were measured with a Zetasizer Nano ZS™ system (Malvern) with a 633 nm laser. CD, UV/vis, and Chirascan-Plus quantitative CD spectra were processed with the OriginLab® software. The temperature was maintained at 25 °C for all measurements, with a scanning range of 200–800 nm under high-purity nitrogen. The UCL spectra were obtained with an FLS980 spectrometer with an external 980 nm laser as the excitation source. Confocal images of cells were acquired with a modified Olympus FV1000 laser scanning upconversion luminescence microscope equipped with a

continuous-wave laser at 980 nm (Connet Fiber Optics, China). The laser provided excitation at 980 nm, and the emitted luminescence was collected at 736 ± 50 nm and 588 ± 50 nm. *In vivo* images of tumors were obtained with the Maestro™ *In-Vivo* Fluorescent Imaging System (CRi Inc.), using a 980 nm optical-fiber-coupled laser as the excitation source.

Supplementary Method

Synthesis of 10nm gold nanoparticles

Water (79 mL) and 1% $HAuCl_4$ (1 mL) were mixed together to produce solution A; 1% sodium citrate (4 mL), 1% gallotannic acid (0.1 mL), and 25 mM K_2CO_3 (0.1 mL) were added to 15.8 mL of water to produce solution B. Solutions A and B were both heated to 60 °C and then solution B was quickly added to solution A with high-speed stirring. The mixture was maintained for 2 h at 60 °C until the color did not change further, and was then cooled to room temperature.

The solution was centrifuged at 13,000 xg for 10 min to concentrate it 10-fold. The supernatant was removed, and the pellet was resuspended in 10 mM Tris-HCl (pH 7.5).

Synthesis of shells modified with GSH

Gold nanoparticles (10 nm, 2 mL) were added to 20 mL of water, and then

30 mg of sodium citrate and 10 mg of silver nitrate were added. Ascorbic acid (2 mg/mL, 1 mL) was added dropwise until the solution turned yellow. The solution was centrifuged at 9000 xg for 10 min, and the pellet was resuspended in 10 mM Tris-HCl (pH 7.5).

GSH solution was added to the solution, to a final concentration of 5 μM. After 4 h, the mixture was centrifuged at 9000 xg for 10 min, and the resulting pellet was resuspended in 5 mg/mL poly(vinylpyrrolidone).

An aqueous solution of 1 mM $HAuCl_4$ (3.5 mL) was added dropwise to the solution with vigorous stirring, and the color of the solution turned blue-violet, after which 200 μL of ascorbic acid (2mg/mL) was added. The solution was centrifuged at 9000 xg for 10 min and resuspended in 2 mL of 10 mM Tris-HCl (pH 7.5).

YSNPs modified with peptide

YSNP (10 nM) was modified with the peptide by mixing both together so that the final concentration of peptide was 5 μM. After incubation for 3 h, the solution was centrifuged at 9000 xg for 10 min to remove any unbound peptide.

Western blotting analysis

In the western blotting analysis, cells were incubated with chiral UYTe for 12 h. Then the cells were lysed with RIPA Lysis Buffer (Beyotime). The protein lysates were washed with cold PBS and the concentrations of protein were separated with sodium dodecyl sulfate polyacrylamide gel electrophoresis and then transferred to polyvinylidene difluoride membranes (Life Technologies). The membranes were incubated with primary antibody LC3 antibody (1:500 dilution), and then with a horseradish-peroxidase-conjugated secondary (goat anti-rabbit) antibody (1:1000 dilution). Tubulin (1:1000 dilution) was used as the loading control.

Histopathological examination

The D-GSH-modified UYTe assembly (200 μL; amount of Au, 2 mg/mL) was injected through the tail veins of the mice. After 24 h, the tumors were harvested and fixed in 10% formalin solution for 12 h. The prepared tumor samples were refrigerated, sliced into 5 μm sections, and mounted on glass slides. Images of the treated tumor sections were obtained with confocal fluorescence microscopy.

Supplementary tables

Supplementary Table 1. The detailed DNA sequence applied in this experiment

**DNA1**	**5'-NH2-GCTTAGCAGTAACGTAGCTGATAGTTAGGCACAGAATCGGTACAGACCGTTGCGGAATCAGCTCACGATTTCCTGATCCTGAATGCATGCTTGATAGCCTAGGTACAATCGCGATCTAAGCCTGGACTCAGGTCGAGCCTTTGGCATGAATCGATCCGATAGCTACGACTGATGCACTTCAGCTCCAATCCGTAACTAGCAGGACGTAGC**
**DNA2**	**5'-SH-CGTATGCCTAGTATAGCAATCGATGGCACTAGCGATGCATCAGCGCTGAGCATAATCCGATAGCTTCCTTTCGAATCGTCATTGCATCGACTATCAATCCGTGTCTTAGCCATGTCTGGCAACGCCTTAGTCGAGTGCTTTTGTAGAGCGTGCATCTGCGTACGAGTGCTGCATCCAGCATGCTATGCGCAAC**
**DNA3-1**	**5'-GATCTGCTTCGGATAACCTATTCCGCCATTTGCATACGGATCATATCGTTAGCTACCGTGATCGCTTTAGTTACATCGATATG**
**DNA3-2**	**5'-CGTAGTCGCGACTCGTATTAGGCTATCGAAGGTTTGCTACGTCCTGCTAGTTACGGATTGGAGCTGAAGTGCATCAGTCGTAGCTATCGGATCGATTCATGCC**
**DNA3-3**	**5'-SH-CACATCGTCACTAAGCAGCTGTGATTCGACGGATACGCGTTTAGTTACATCGATATG**
**DNA4**	**5'-SH-GTTGCGCATAGCATGCTGGATGCAGCACTCGTACGCAGATGCACGCTCTACCGGTATAGCTACATTGATTTCTAGACGAAGCCTATTGGATAAGGCGGTATCGCGTATCCGTCGAATCACAGCTGCTTAGTGACGATGTGTTTGGCTCGACCTGAGTCCAGGCTTAGATCGCGATTGTACCTAGGCTATCAAGCATGCATTCAGGATCAGG**
**ATP-apt**	**SH-CATATCACCTGGGGGAGTATTGCGGAGGAAGGTGTAACT**

Supplementary Table 2. The fitting data of SAXS spectrum

**Sample**	**r**	**Dmax**
YSNPs	11.0	62.18
UYTe	10.3	100
YSNP-YSNP-UCNP trimer	13.59	102

Supplementary figures

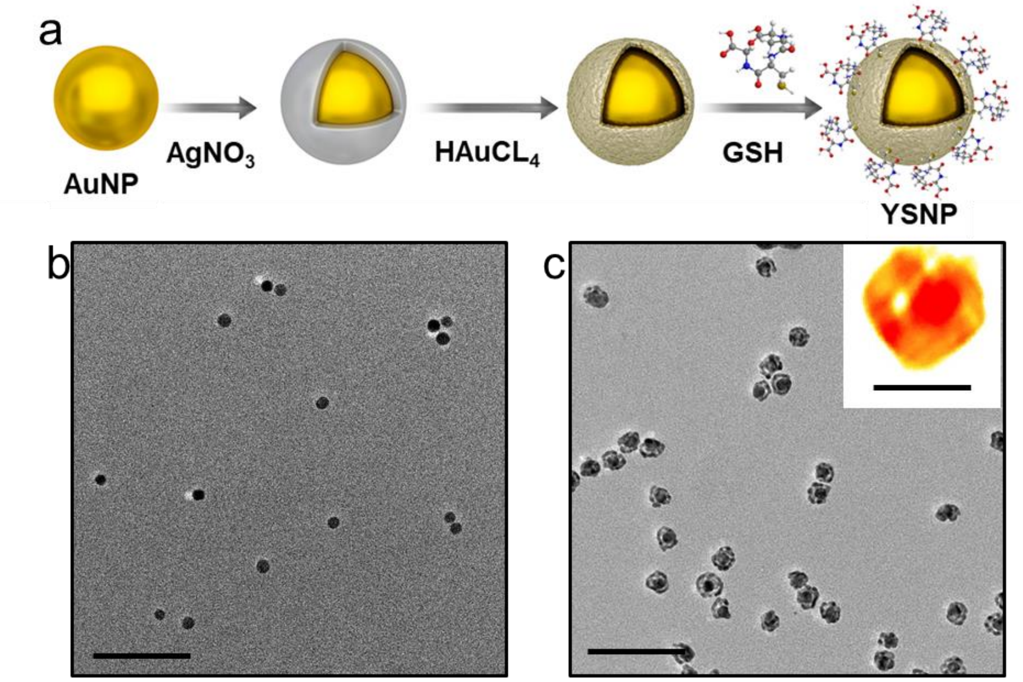

Supplementary Figure 1. (a) The fabrication of YSNP from AuNP. Typical TEM images of UCNPs and YSNPs used in this project: (b), 10 ± 2 nm UCNPs; (c), 20 ± 3nm YSNPs, scale bar 100 nm. Inset shows the cross-section 3D reconstruction cryo-TEM tomography image of YSNP, scale bar 20 nm. All experiments were performed in triplicate.

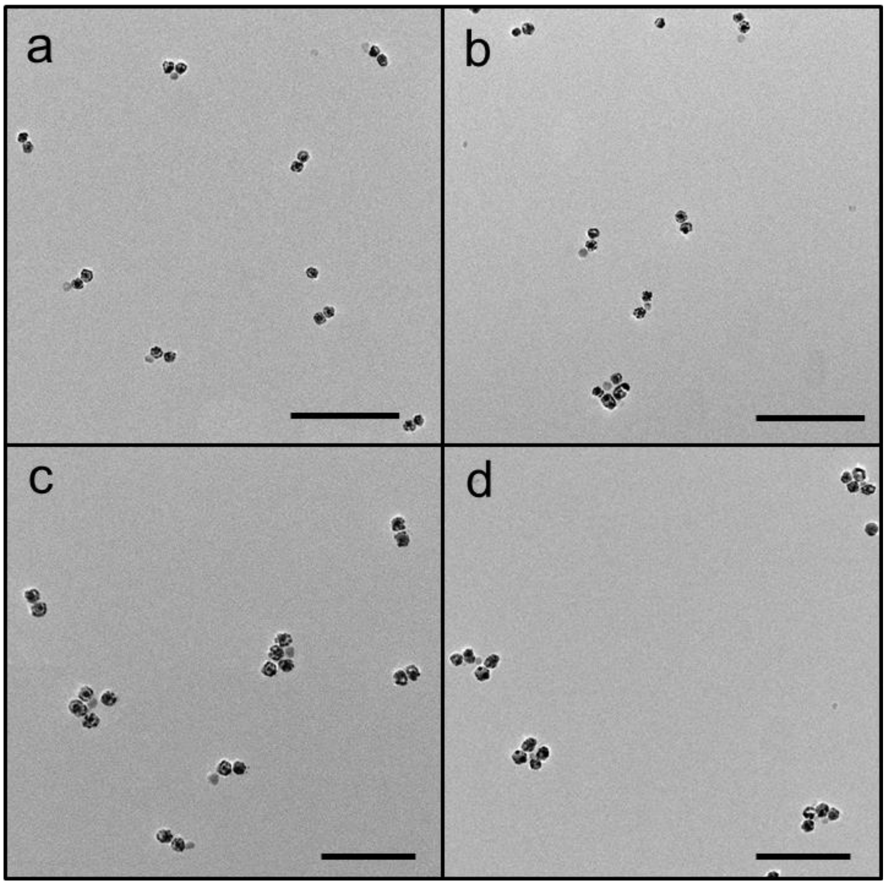

Supplementary Figure 2. Representative TEM images of the assembled UYTe nanostructures at different assembly times: (a) 0h; (b) 3h; (c) 6h; (d) 9h, scale bar 200 nm. All experiments were performed in triplicate.

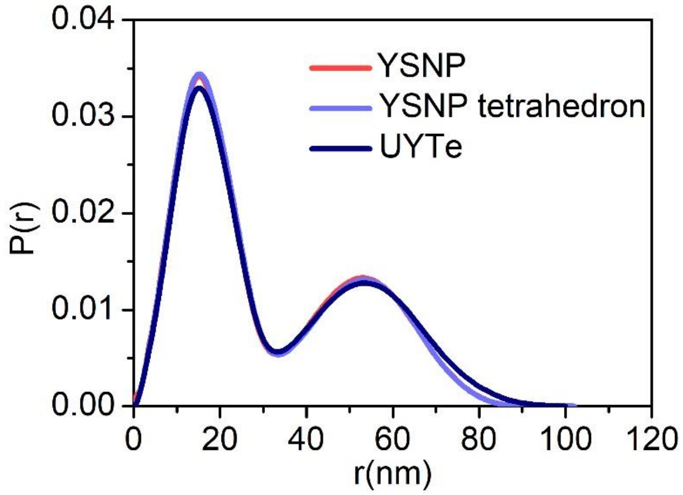

Supplementary Figure 3. The P(r)-derived radii and the maximum intraparticle distance (D_{max}) were obtained using GNOM. All experiments were performed in triplicate.

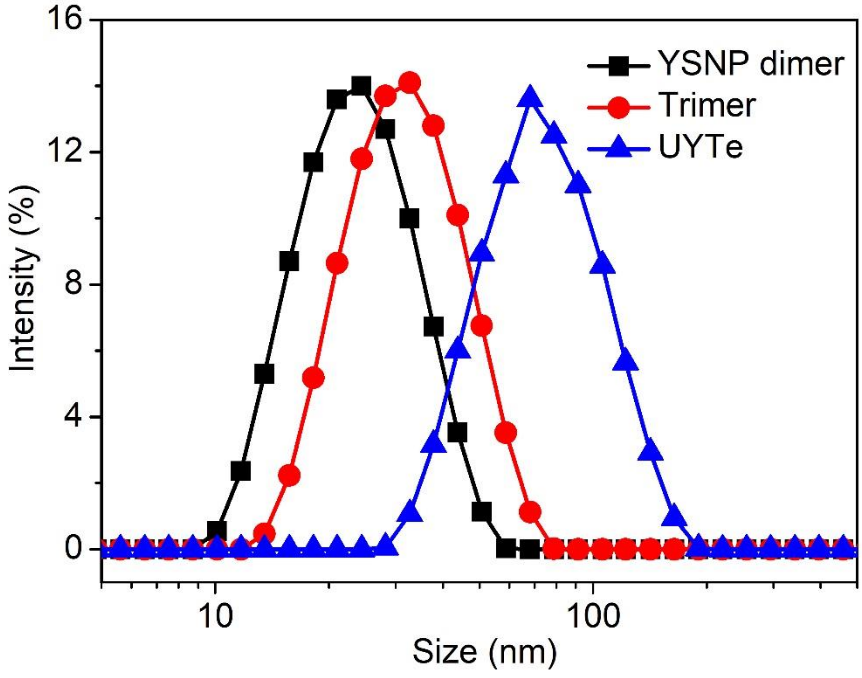

Supplementary Figure 4. The DLS spectra of YSNP dimer, YSNP-YSNP-UCNP trimer, UYTe nanostructures. All experiments were performed in triplicate.

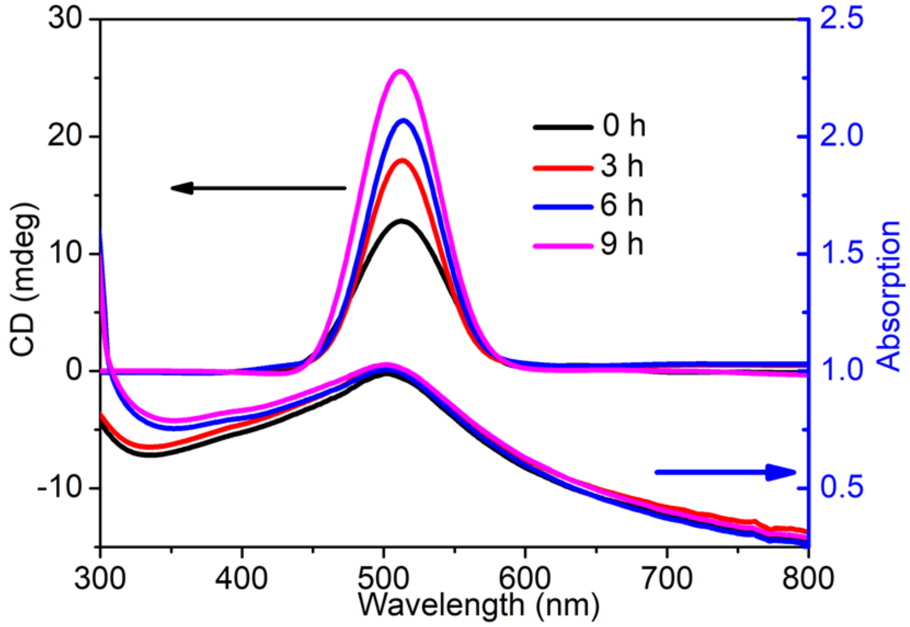

Supplementary Figure 5. The CD and UV-vis absorption spectra of UYTe nanostructures acquired at different assembly times. All experiments were performed in triplicate.

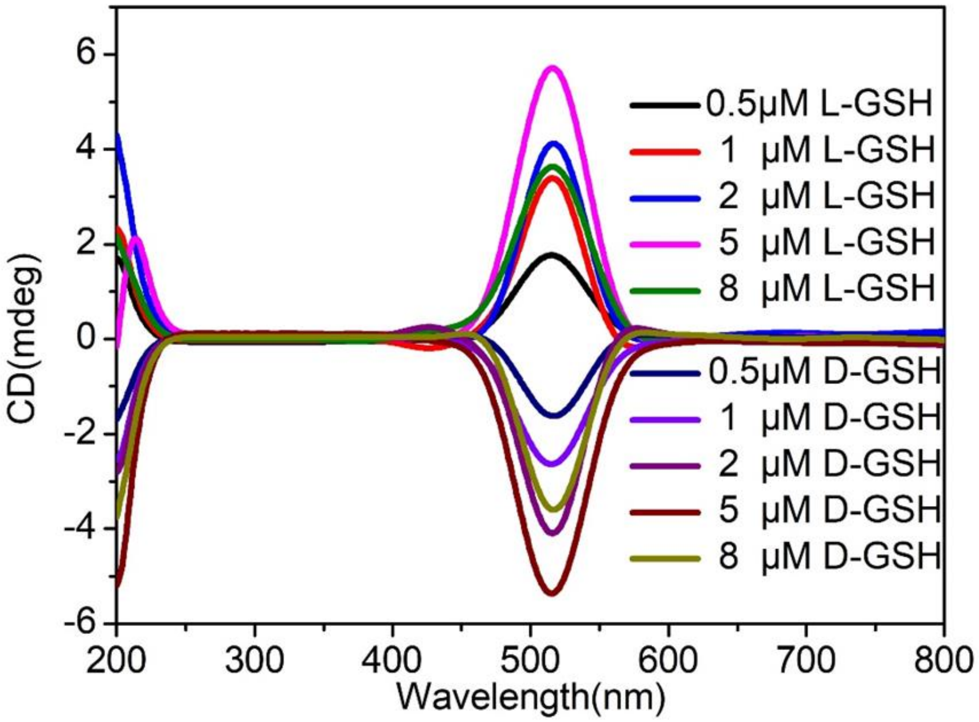

Supplementary Figure 6. The CD spectra of YSNPs modified with different concentration of D-/ L- GSH. All experiments were performed in triplicate.

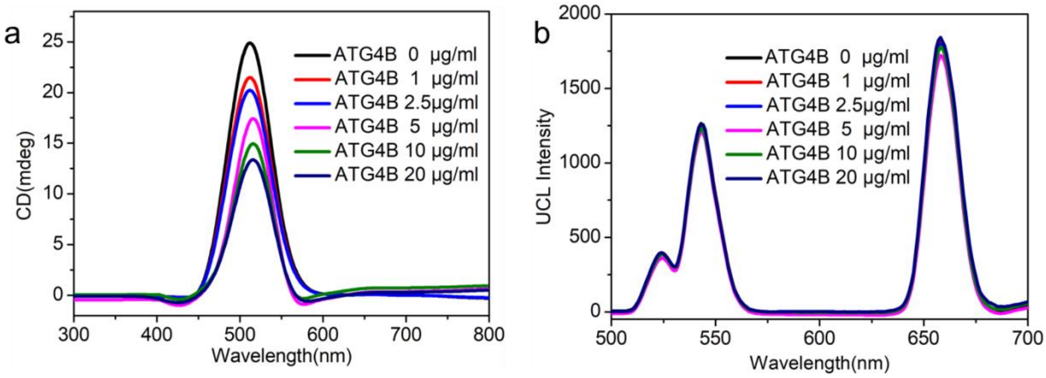

Supplementary Figure 7. The (a) CD and (b) UCL spectrum of UYTe nanostructures incubated with different concentration of ATG4B (autophagy-specific enzyme). All experiments were performed in triplicate.

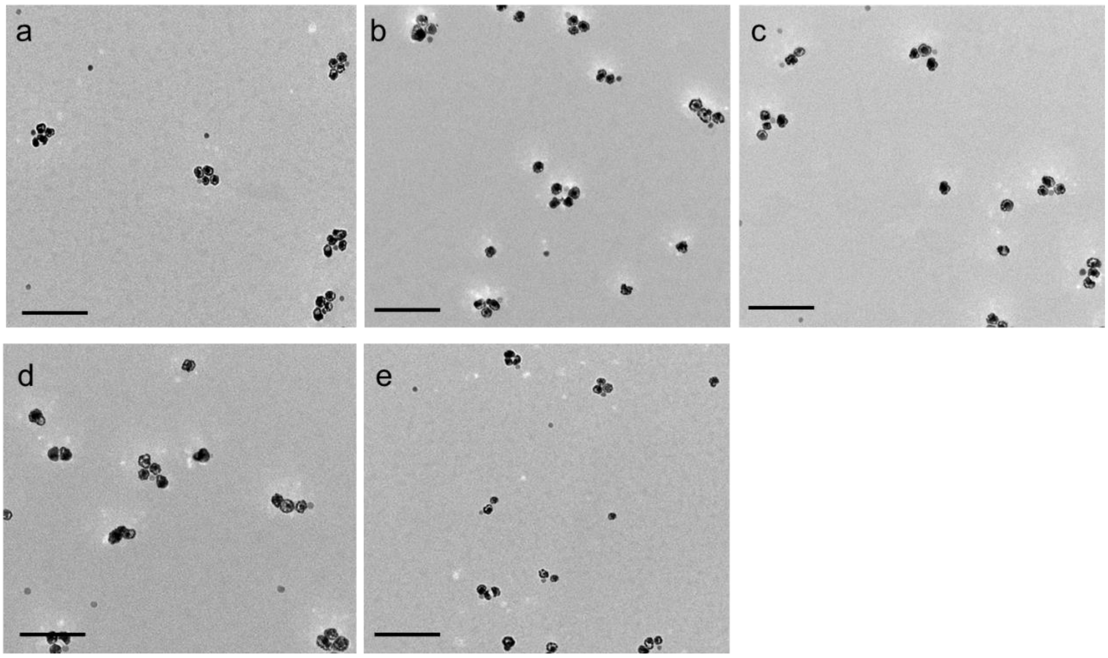

Supplementary Figure 8. The TEM images of UYTe nanostructures incubated with different concentration of ATG4B (autophagy-specific enzyme) (a) 0 μg/mL (b) 2.5 μg/mL, (c) 5 μg/mL, (d) 10 μg/mL, (e) 20 μg/mL, scale bar 200 nm. All experiments were performed in triplicate.

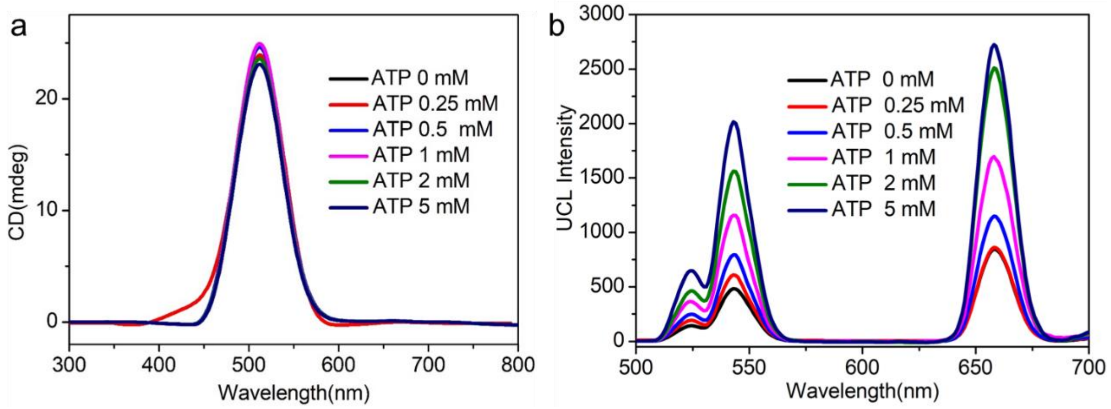

Supplementary Figure 9. The (a) CD and (b) UCL spectrum of UYTe nanostructures incubated with different concentration of ATP. All experiments were performed in triplicate.

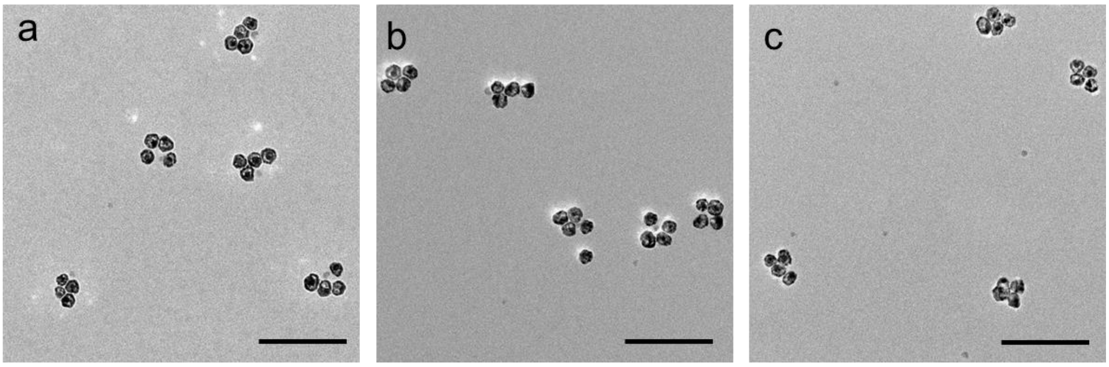

Supplementary Figure 10. The TEM images of UYTe nanostructures incubated with different concentration of ATP (a) 0mM, (b) 1mM, (c) 5mM, scale bar 100 nm. All experiments were performed in triplicate.

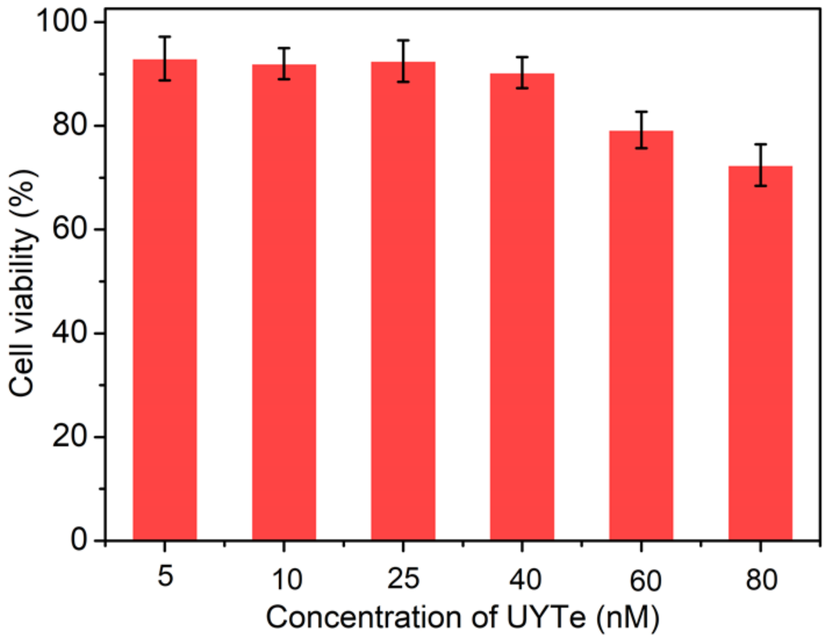

Supplementary Figure 11. The cell viability of MCF-7 after incubated with different concentration of UYTe assembly (without GSH modification) for 24h. The data are shown as the mean ± s.d. (n = 3).

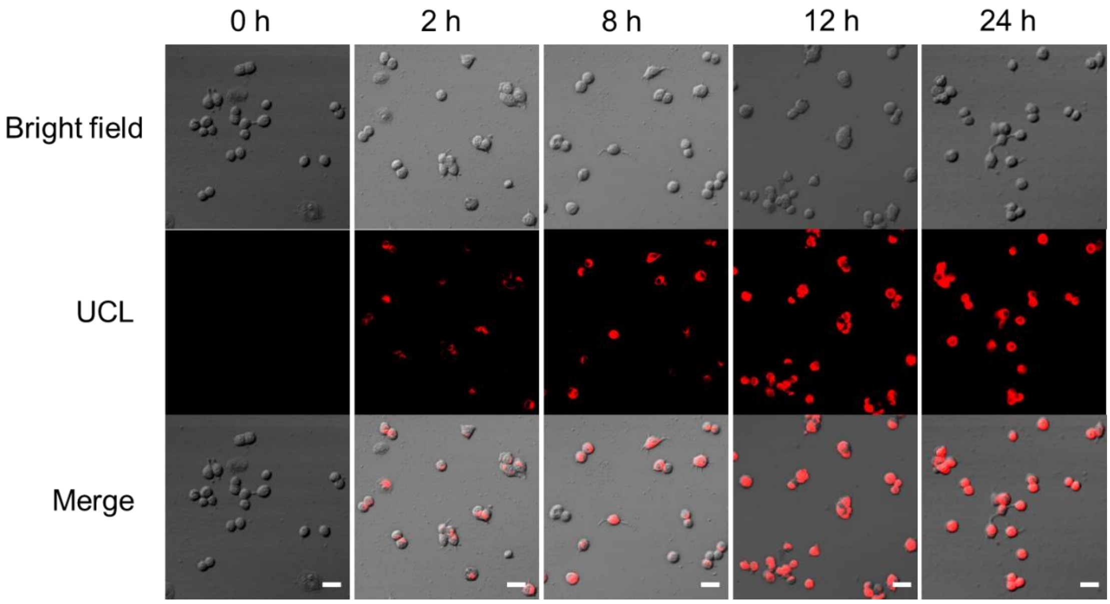

Supplementary Figure 12. The confocal images of MCF-7 cells (5×10^7) incubated UYTe (without GSH modification) for 0-24 h. Scale bar 20 μm. All experiments were performed in triplicate.

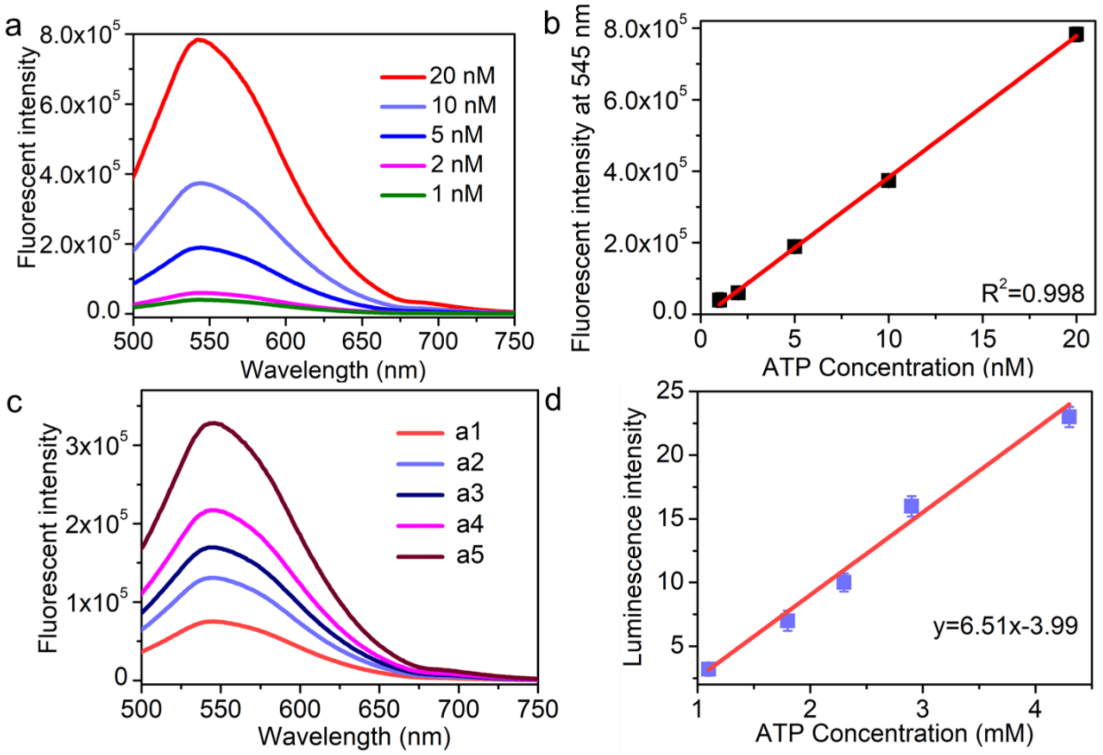

Supplementary Figure 13. The (a) fluorescent intensity of ATP detection kit after incubated with different concentration of ATP. (b) Plot of the fluorescent intensity at 545nm of ATP detection kit versus the different concentration of ATP. (c) The corresponding intracellular ATP concentration in **Fig. 4 (a)** detected by ATP kit. (a1) 20 μg mL^{-1} oligomycin, (a2) 10 μg mL^{-1} oligomycin, (a3) PBS, (a4) 50 μg mL^{-1} etoposide, (a5) 100 μg mL^{-1} etoposide. (d) Plot of luminescence intensity of UYTe versus the different intracellular ATP concentration. All experiments were performed in triplicate. The data are shown as the mean ± s.d. (n = 3).

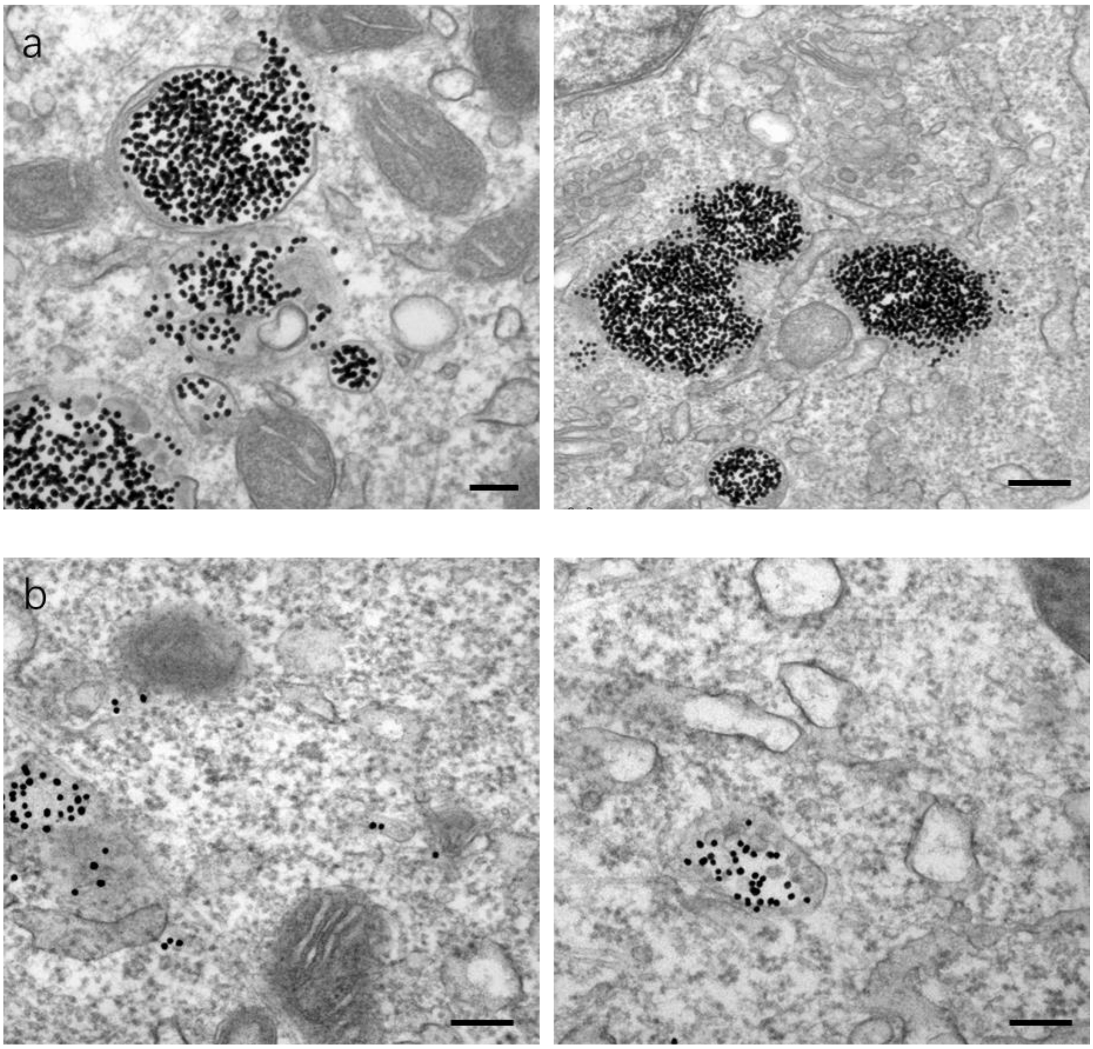

Supplementary Figure 14. The bio-TEM images of MCF-7 cells (5×10^7) treated by (a) D-GSH modified UYTe (40 nM), and (b) L-GSH modified UYTe (40 nM) for 12h, scale bar 200 nm. All experiments were performed in triplicate.

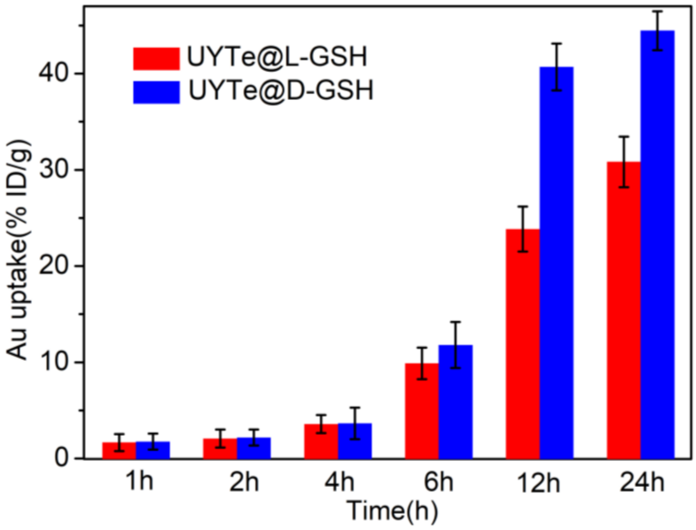

Supplementary Figure 15. The intracellular bio-distribution of Au amounts after 24h treatment of D- / L-GSH modified UYTe were measured by ICP-MS. The data are shown as the mean ± s.d. (n = 3).

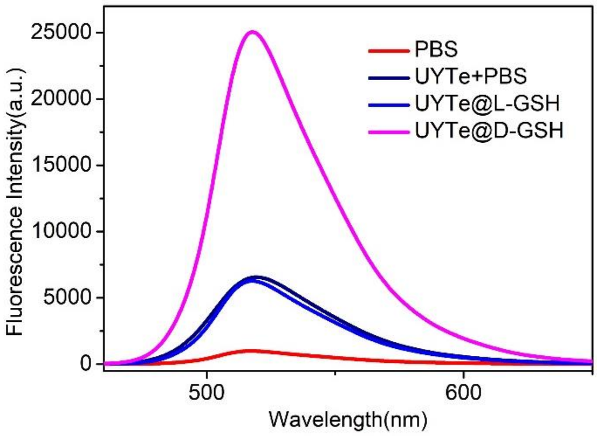

Supplementary Figure 16. The ROS production of MCF-7 cells (5×10^7) treated by PBS, UYTe (40 nM) in PBS, L-GSH modified UYTe (40 nM), and D-GSH modified UYTe (40 nM) for 12h measured by H2DCFDA (Invitrogen™) (GSH concentration is 5 μM). All experiments were performed in triplicate.

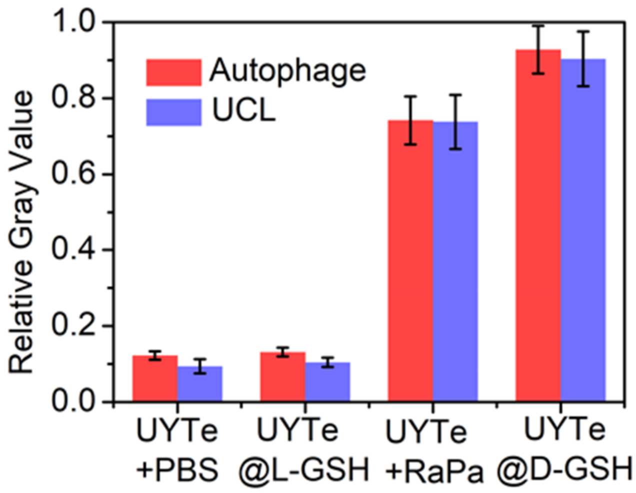

Supplementary Figure 17. The corresponding (d) relative gray value statistic of MCF-7 cells in **Figure 4(b)**. The data are shown as the mean ± s.d. (n = 3).

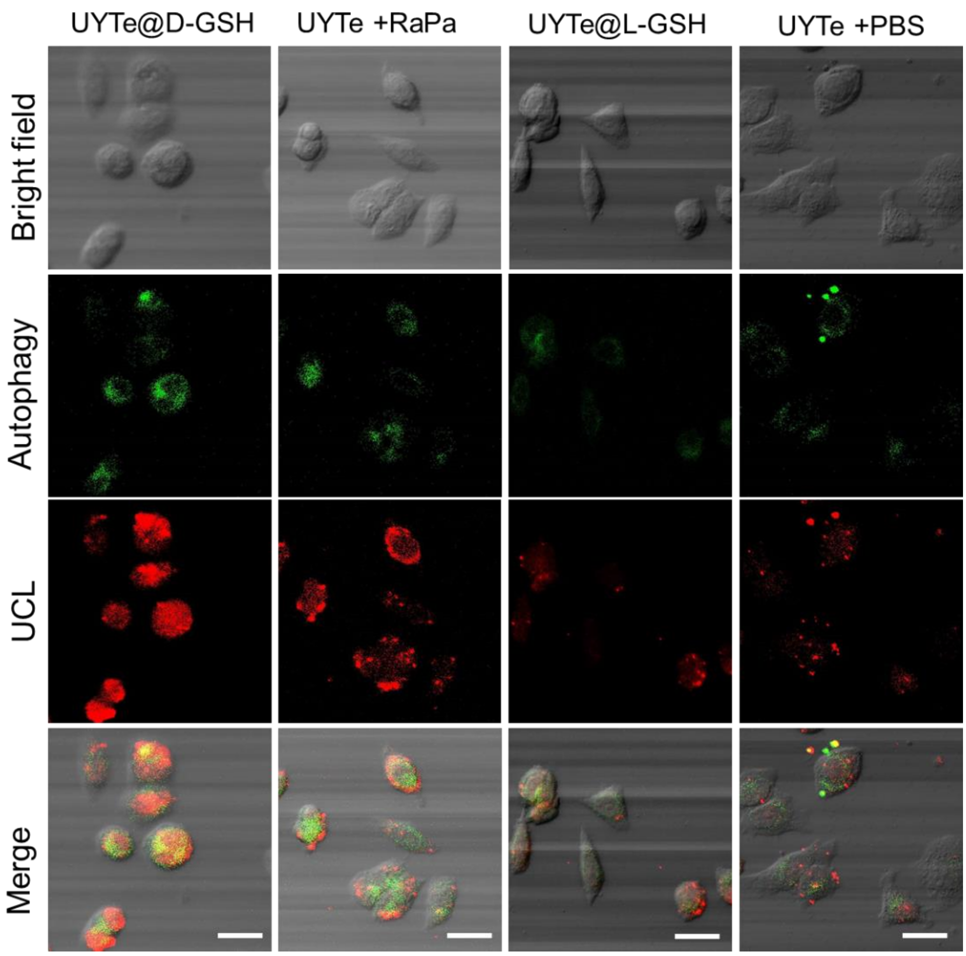

Supplementary Figure 18. The confocal images of MCF-7 cells (5×10^7) treated by UYTe (40 nM) with PBS, L-GSH modified UYTe (40 nM), UYTe (40 nM) with Rapamycin (autophagy inducer 10 μM) and D-GSH modified UYTe (40 nM) 12h after thymine treatment. Scale bar 20 μm. All experiments were performed in triplicate.

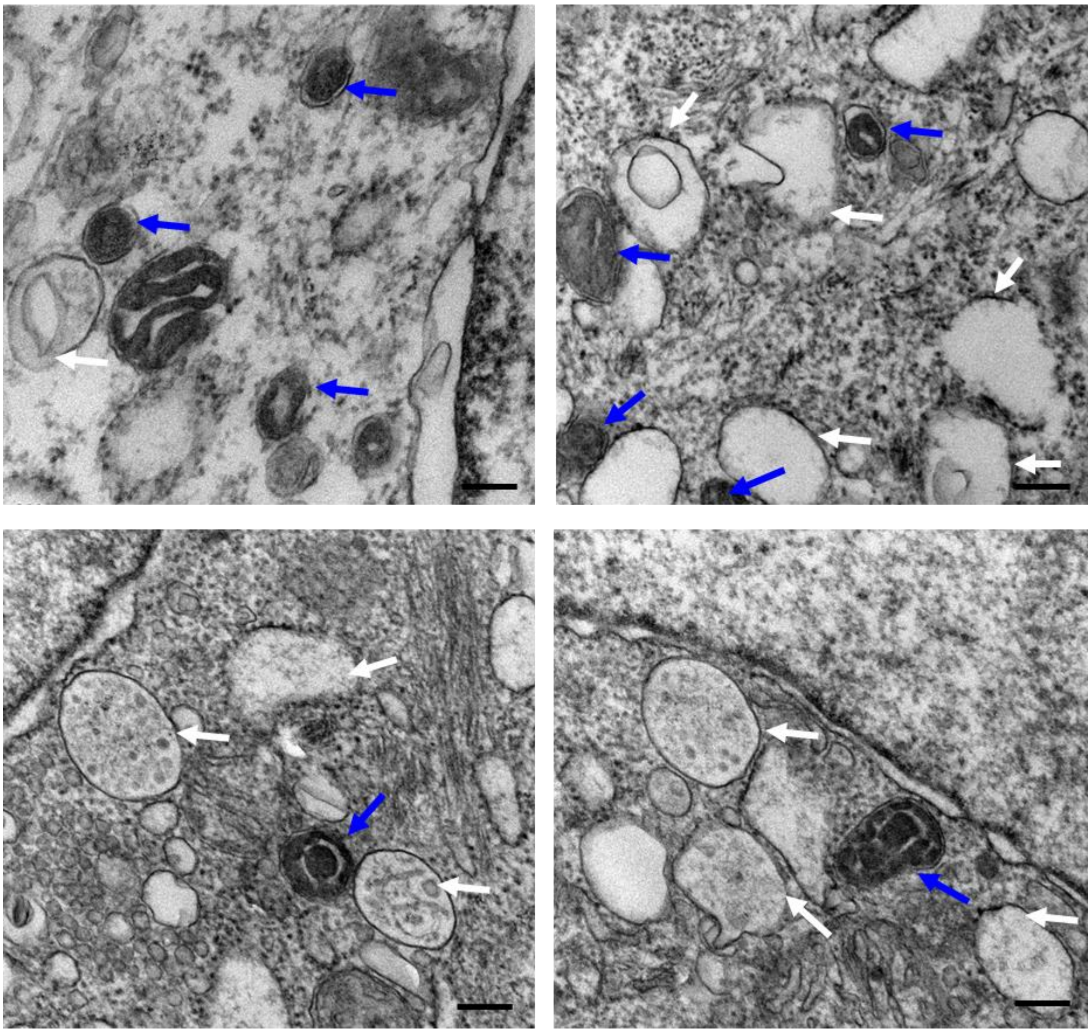

Supplementary Figure 19. Enlarged bio-TEM images of MCF-7 cells (5×10^7) treated by UYTe (40 nM) with Rapamycin (autophagy inducer 10 μM) (40 nM) for 12h. The blue arrow indicates the autophagosomes and the white arrow means the autolysosomes, scale bar 200 nm. All experiments were performed in triplicate.

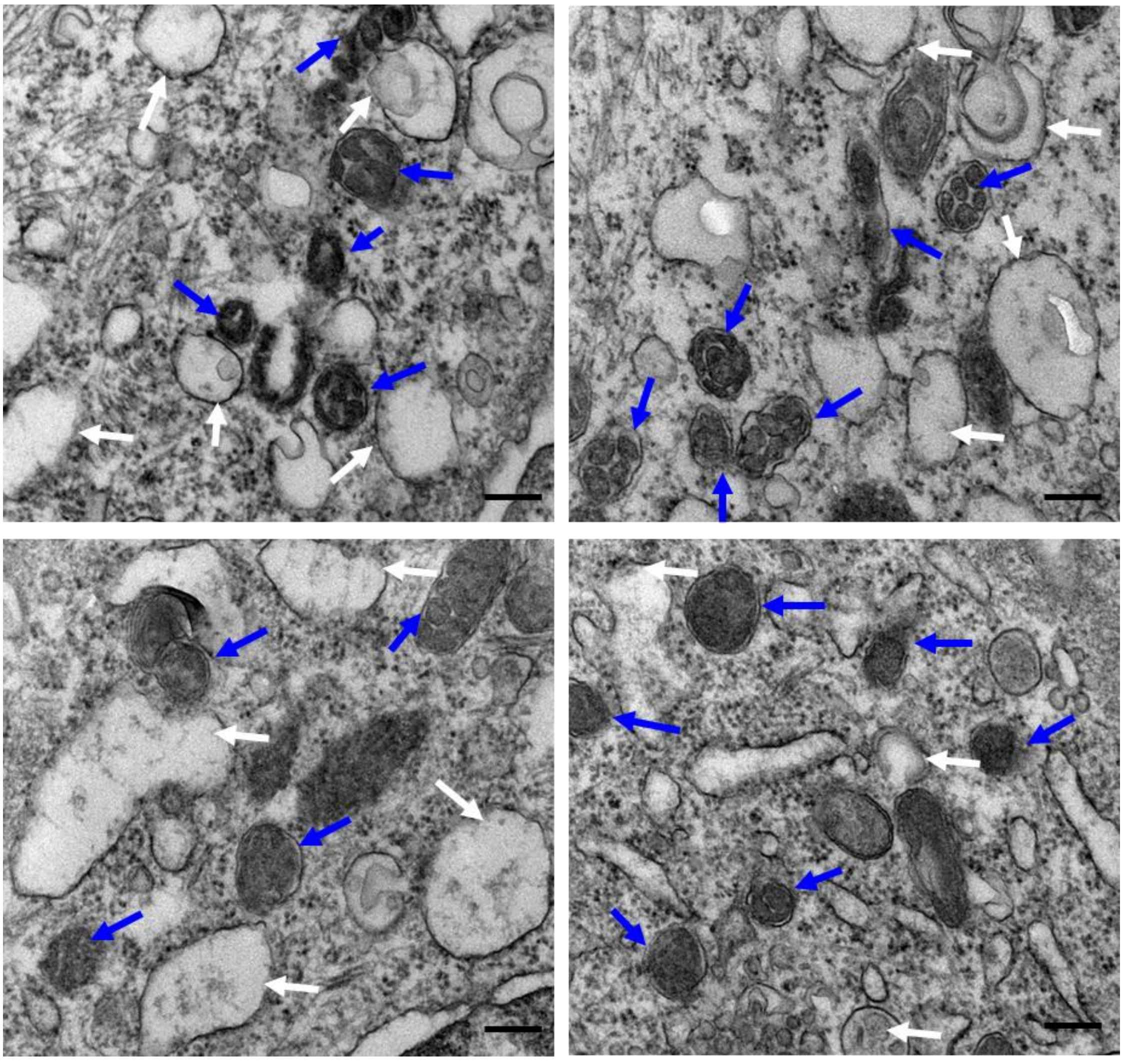

Supplementary Figure 20. Enlarged bio-TEM images of MCF-7 cells (5×10^7) treated by D-GSH modified UYTe (40 nM) for 12h. The blue arrow indicates the autophagosomes and the white arrow means the autolysosomes, scale bar 200 nm. All experiments were performed in triplicate.

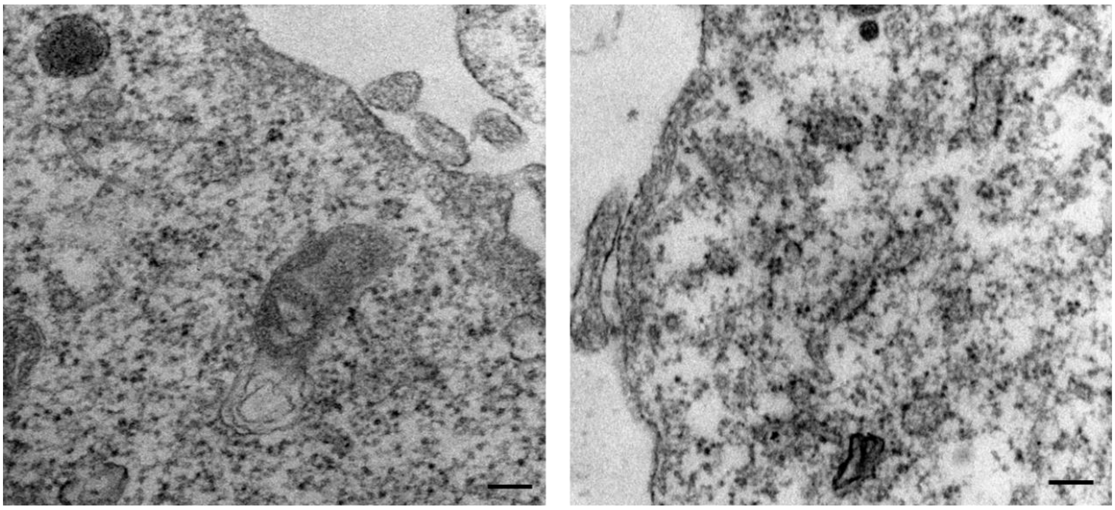

Supplementary Figure 21. Enlarged bio-TEM images of MCF-7 cells (5×10^7) treated by L-GSH modified UYTe (40 nM) for 12h. The blue arrow indicates the autophagosomes and the white arrow means the autolysosomes, scale bar 200 nm. All experiments were performed in triplicate.

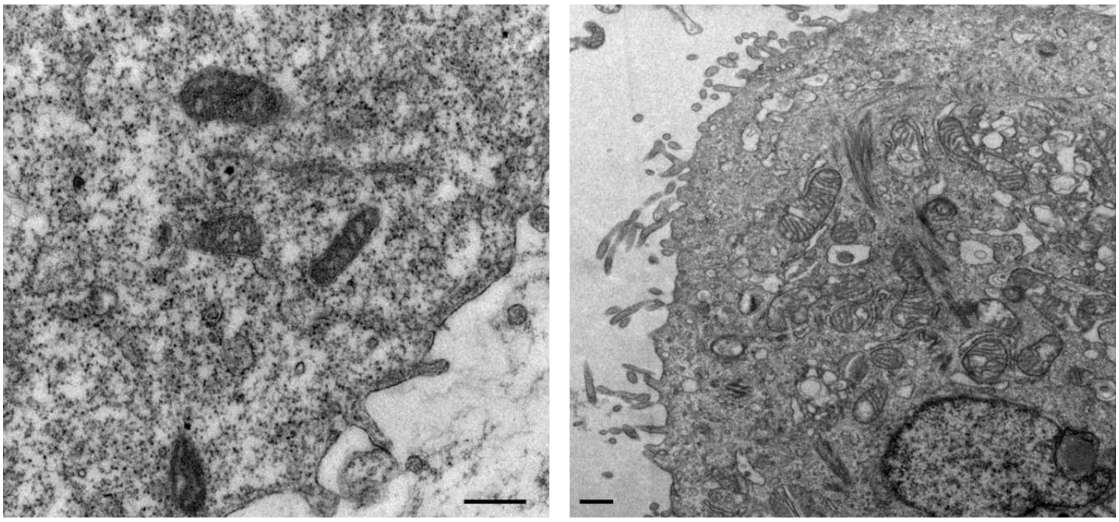

Supplementary Figure 22. Enlarged bio-TEM images of MCF-7 cells (5×10^7) treated by UYTe (40 nM) with PBS for 12h. The blue arrow indicates the autophagosomes and the white arrow means the autolysosomes, scale bar 200 nm. All experiments were performed in triplicate.

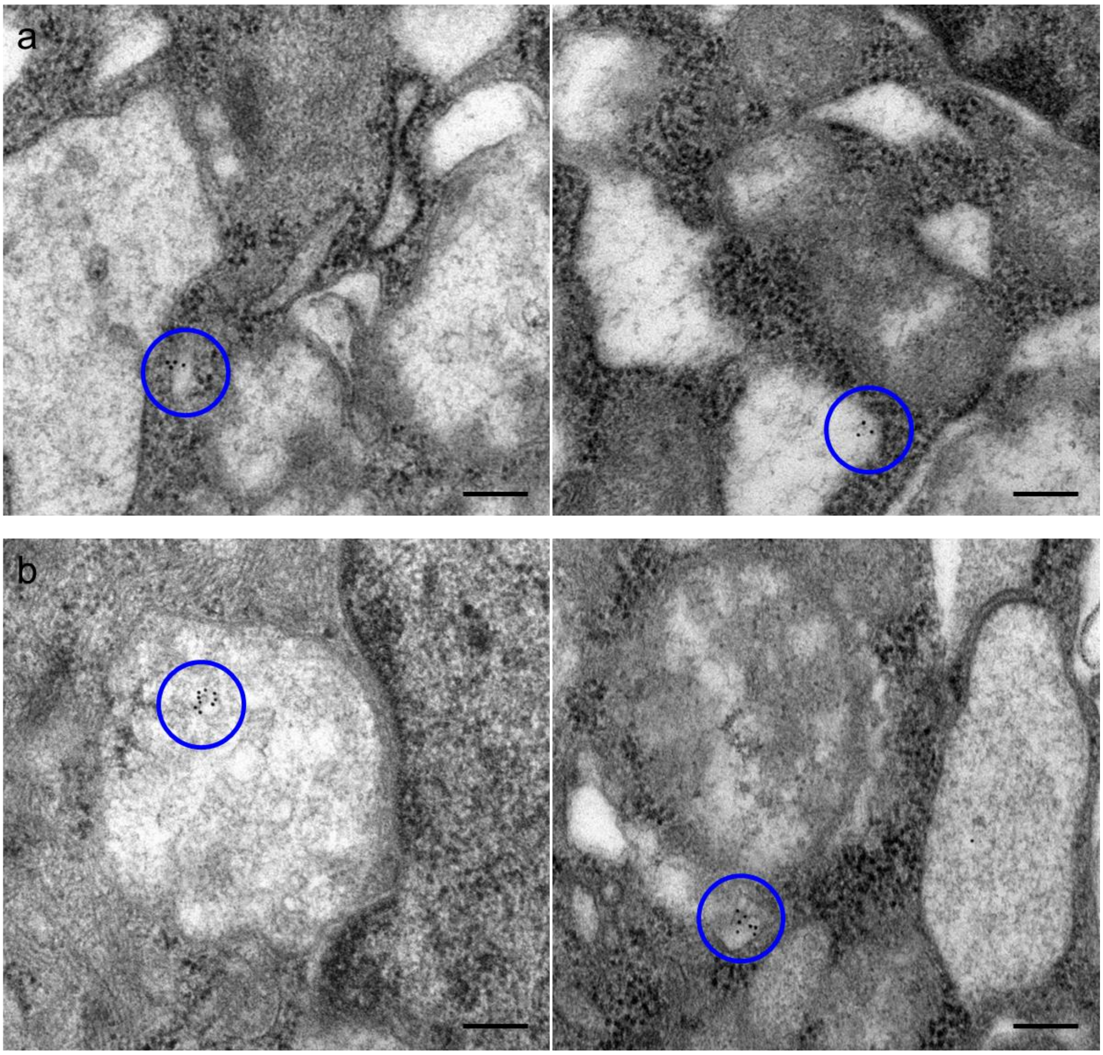

Supplementary Figure 23. Immuno-electron microscope images of the autophagic membranes in MCF-7 cells targeted by LC3-II antibody after (a) D-GSH modified UYTe (b) L-GSH modified UYTe. The blue circle indicates the LC3-II antibody labeled AuNPs, scale bar 200 nm. All experiments were performed in triplicate.

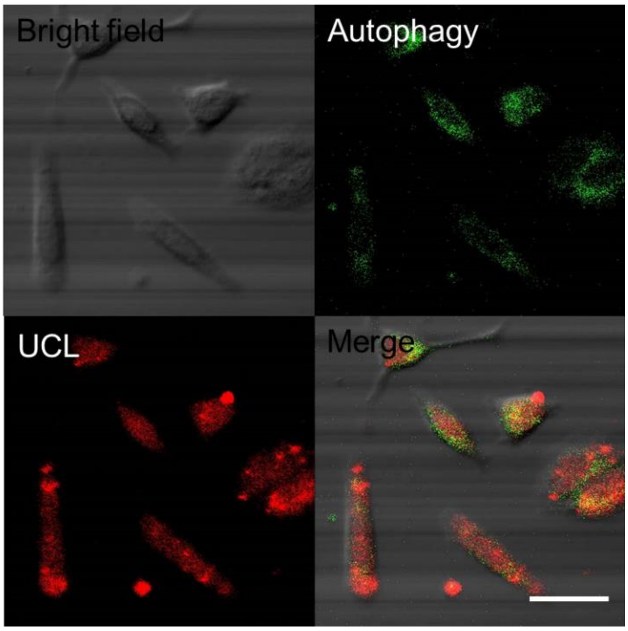

Supplementary Figure 24. The confocal images of MCF-7 cells (5×10^7) treated UYTe without GSH modification (40 nM) for 12 h incubated in the serum starve media. Scale bar 20 μm. All experiments were performed in triplicate.

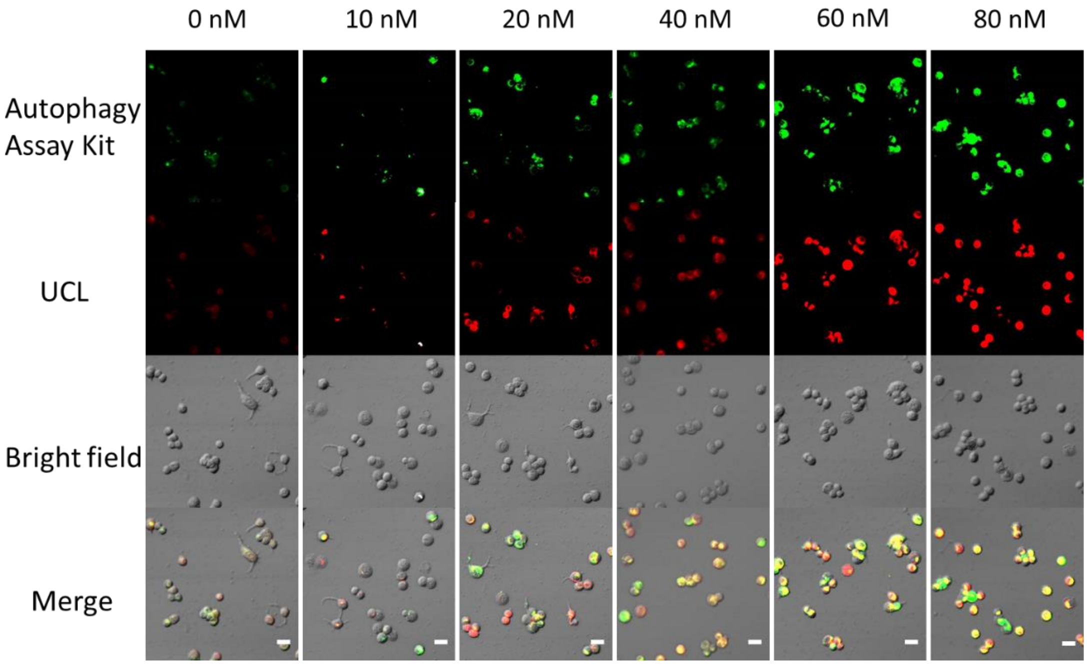

Supplementary Figure 25. The confocal images of MCF-7 cells (5×10^7) treated by different concentration of D-GSH modified UYTe for 12h. Scale bar 20 μm. All experiments were performed in triplicate.

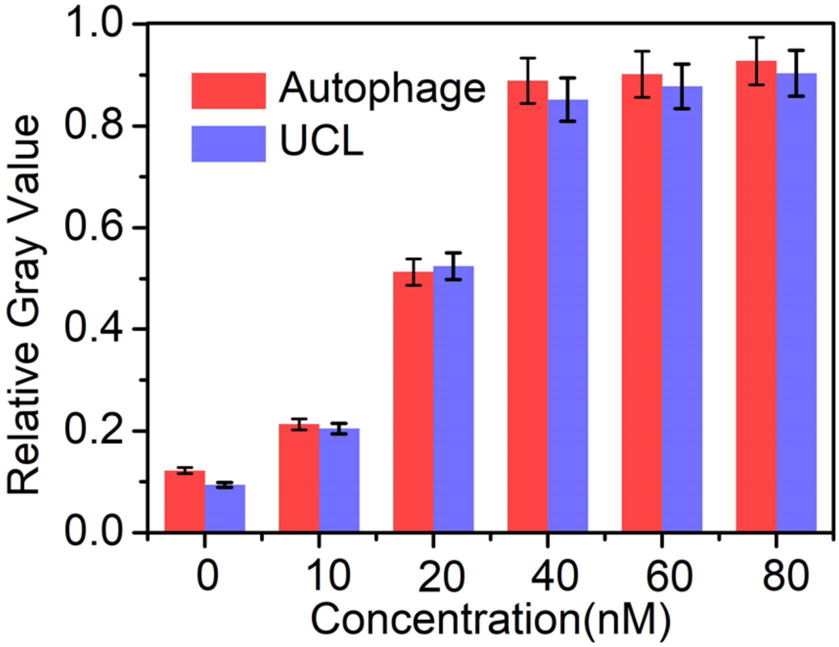

Supplementary Figure 26. The statistics relative gray value of MCF-7 cells (5×10^7) treated by different concentration of D-GSH modified UYTe for 12h. The data are shown as the mean ± s.d. (n = 3).

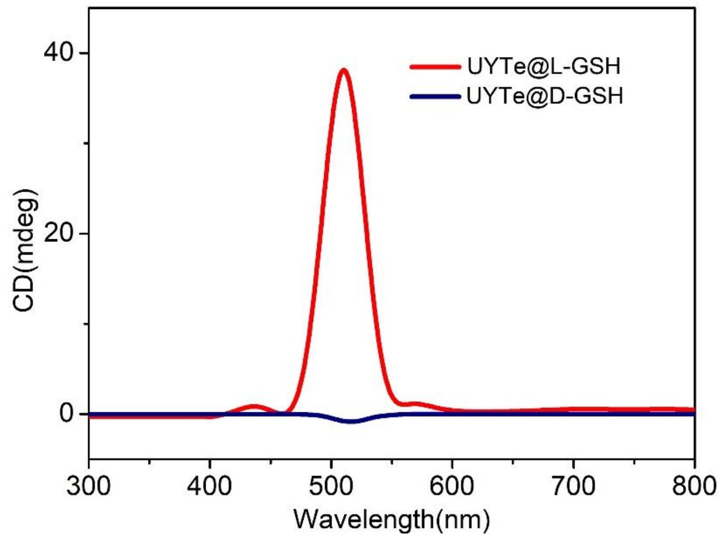

Supplementary Figure 27. The CD spectrum of D-/ L-GSH modified UYTe with 1 μM GSH modification. All experiments were performed in triplicate.

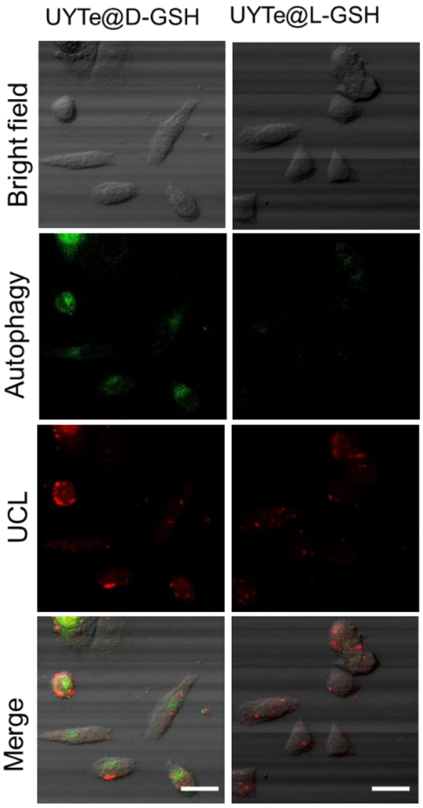

Supplementary Figure 28. The confocal images of MCF-7 cells (5×10^7) treated by L-GSH (1μM) modified UYTe (40 nM) and D-GSH (1μM) modified UYTe (40 nM) for 12h and detected by the Premo™ Autophagy Sensor LC3B-RFP, BacMam 2.0 (Thermo Fisher). Scale bar 20 μm. All experiments were performed in triplicate.

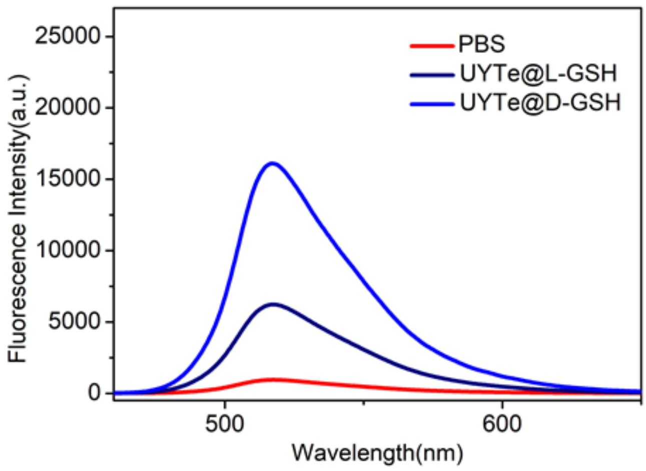

Supplementary Figure 29. The ROS production of MCF-7 cells (5×10^7) treated by PBS, L-GSH (1μM) modified UYTe (40 nM), and D-GSH (1μM) modified UYTe (40 nM) for 12h measured by H2DCFDA (Invitrogen™). All experiments were performed in triplicate.

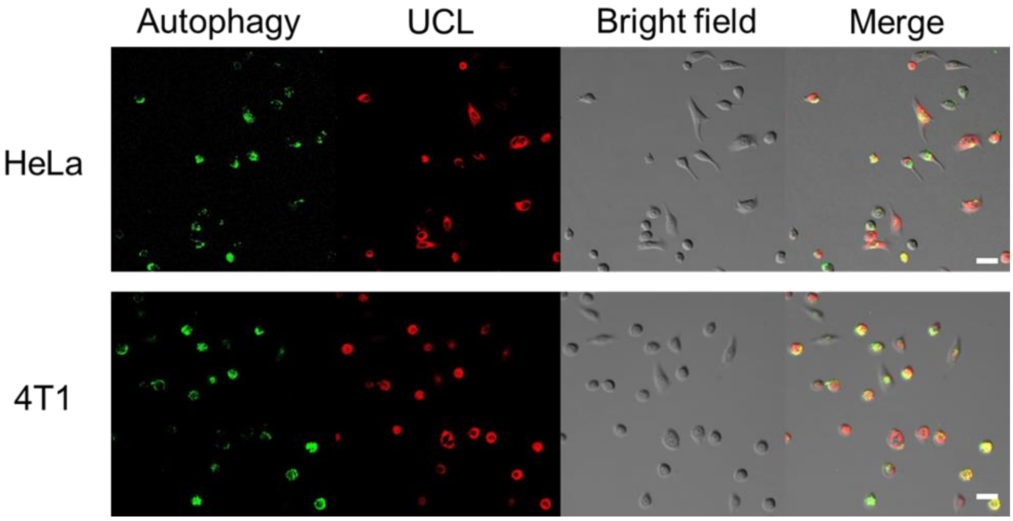

Supplementary Figure 30. The confocal images of (a) HeLa and (b) 4T1 cells (5×10^7) treated by D-GSH modified UYTe for 12h. Scale bar 20 μm. All experiments were performed in triplicate.

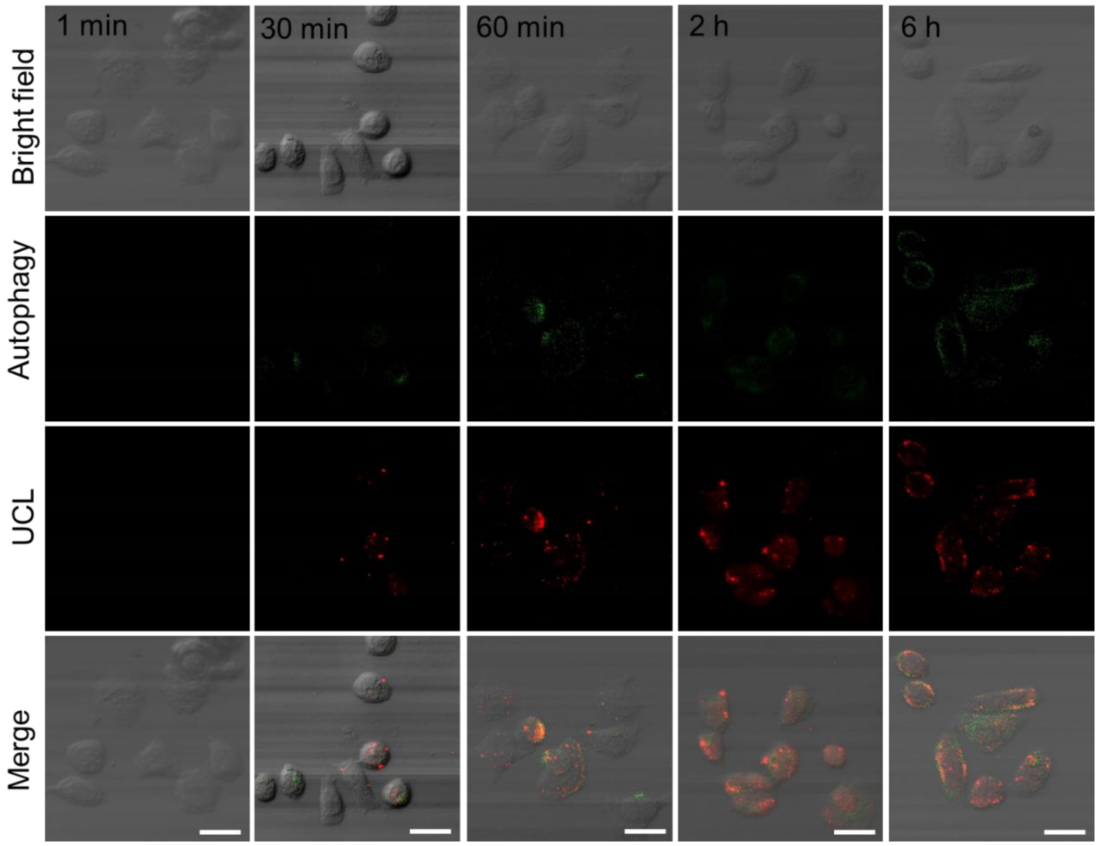

Supplementary Figure 31. The confocal images of MCF-7 cells (5×10^{7}) treated by D-GSH-modified UYTe (40 nM) for different times. Scale bar 20 μm. All experiments were performed in triplicate.

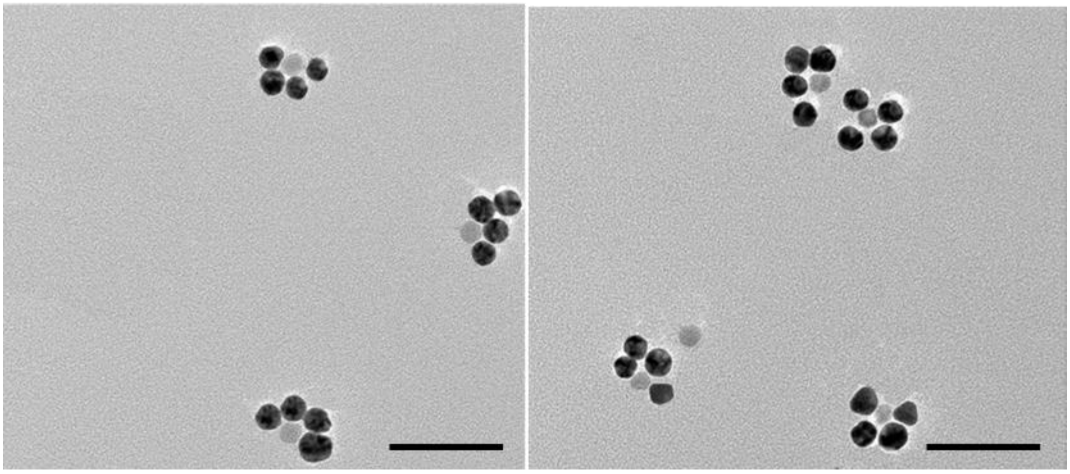

Supplementary Figure 32. The TEM images of UCNP-centered Au NP tetrahedron structure, scale bar 50 nm. All experiments were performed in triplicate.

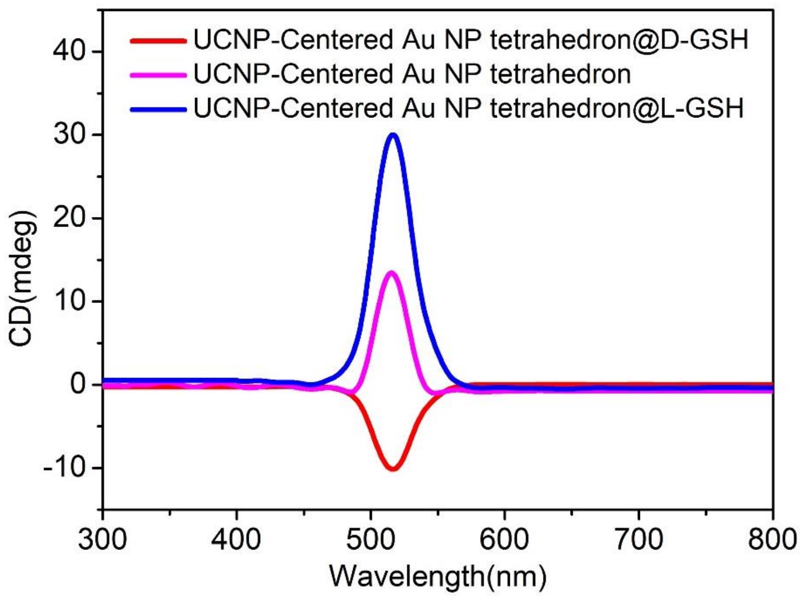

Supplementary Figure 33. The CD spectrum of UCNP-centered Au NP tetrahedron structure before and after D- or L- GSH modification. All experiments were performed in triplicate.

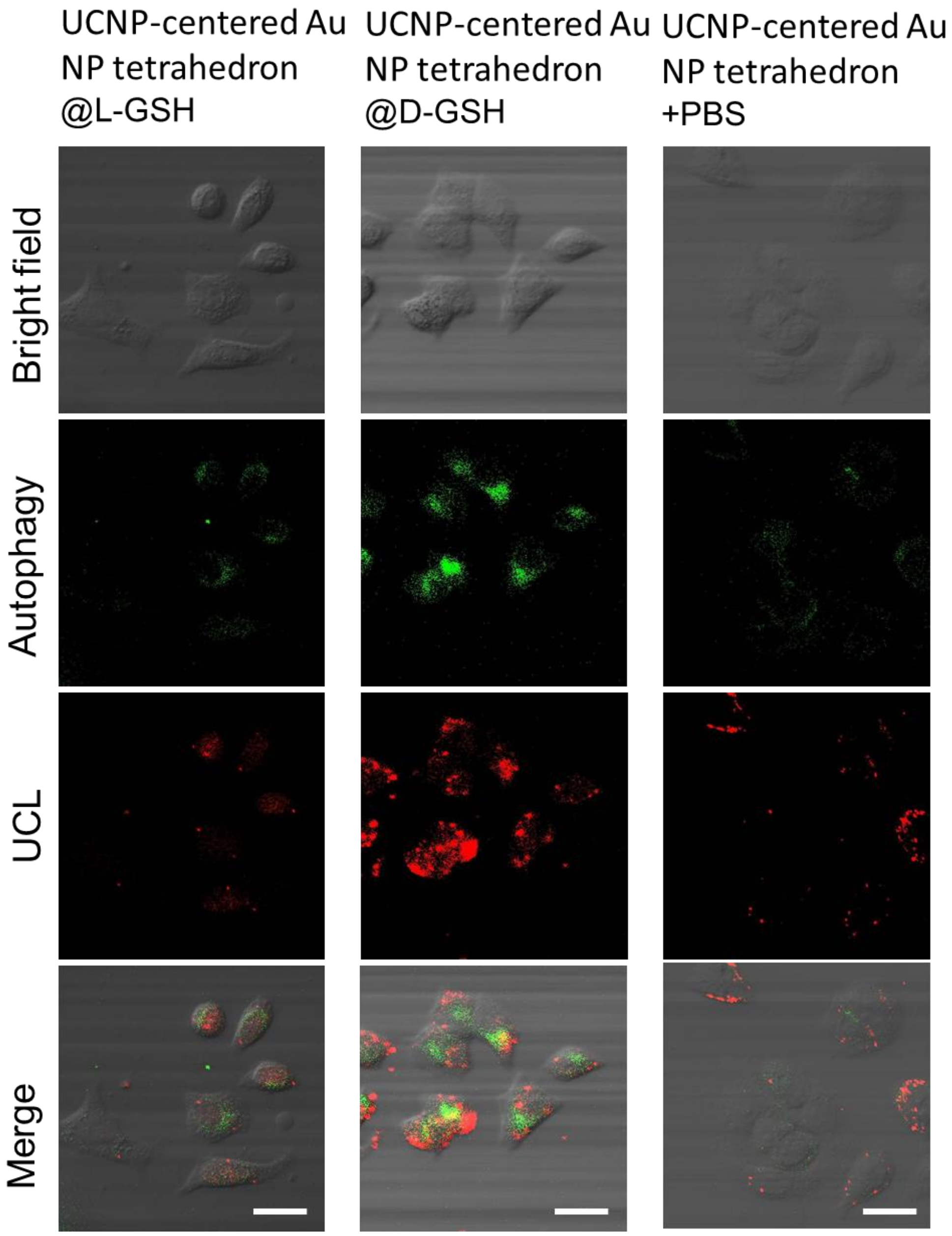

Supplementary Figure 34. The confocal images of MCF-7 cells (5×10^7) treated by UCNP-Centered Au NP tetrahedron (40 nM) with PBS, L-GSH modified UCNP-Centered Au NP tetrahedron (40 nM) and D-GSH modified UCNP-Centered Au NP tetrahedron (40 nM)12h. Scale bar 20 μm. All experiments were performed in triplicate.

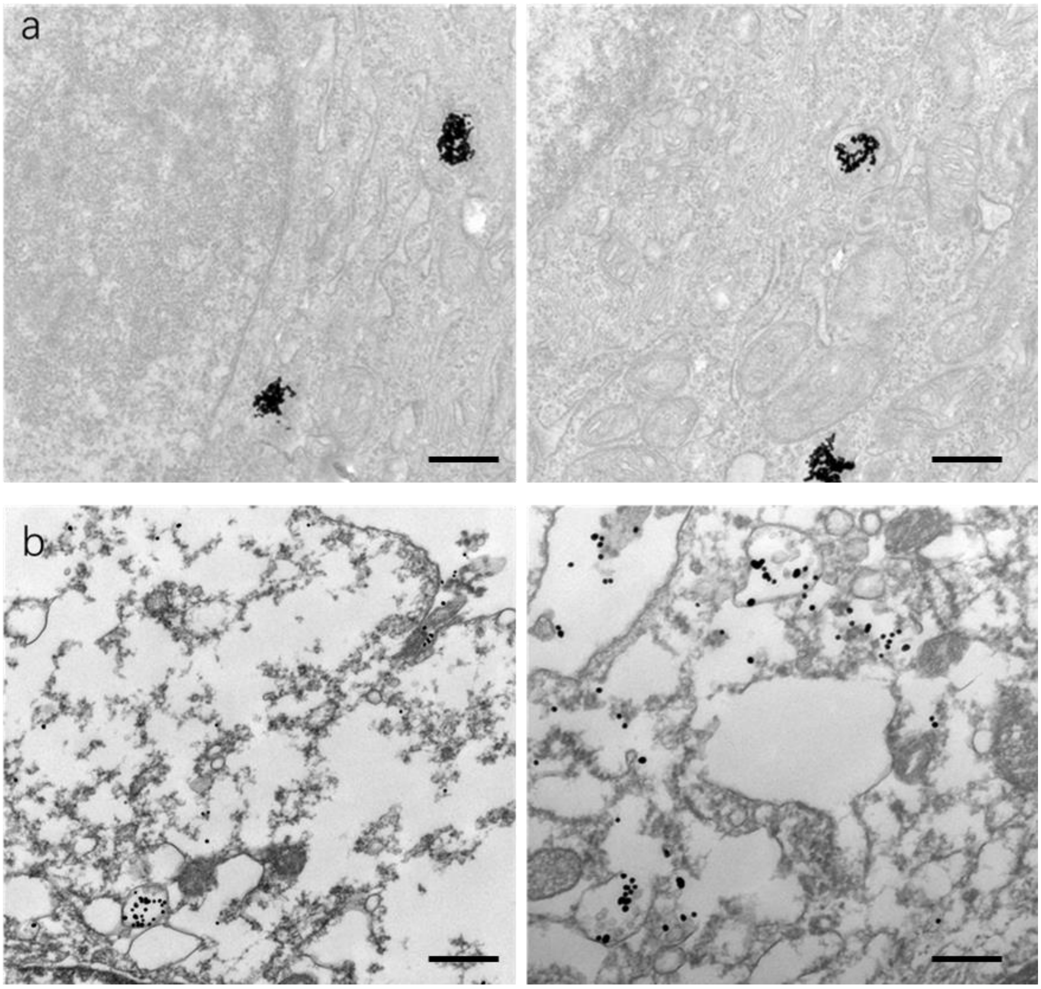

Supplementary Figure 35. The bio-TEM images of MCF-7 cells (5×10^7) treated by (a) D-GSH modified UCNP-Centered Au NP tetrahedron (40 nM), (b) L-GSH modified UCNP-Centered Au NP tetrahedron (40 nM)12h, scale bar 200 nm. All experiments were performed in triplicate.

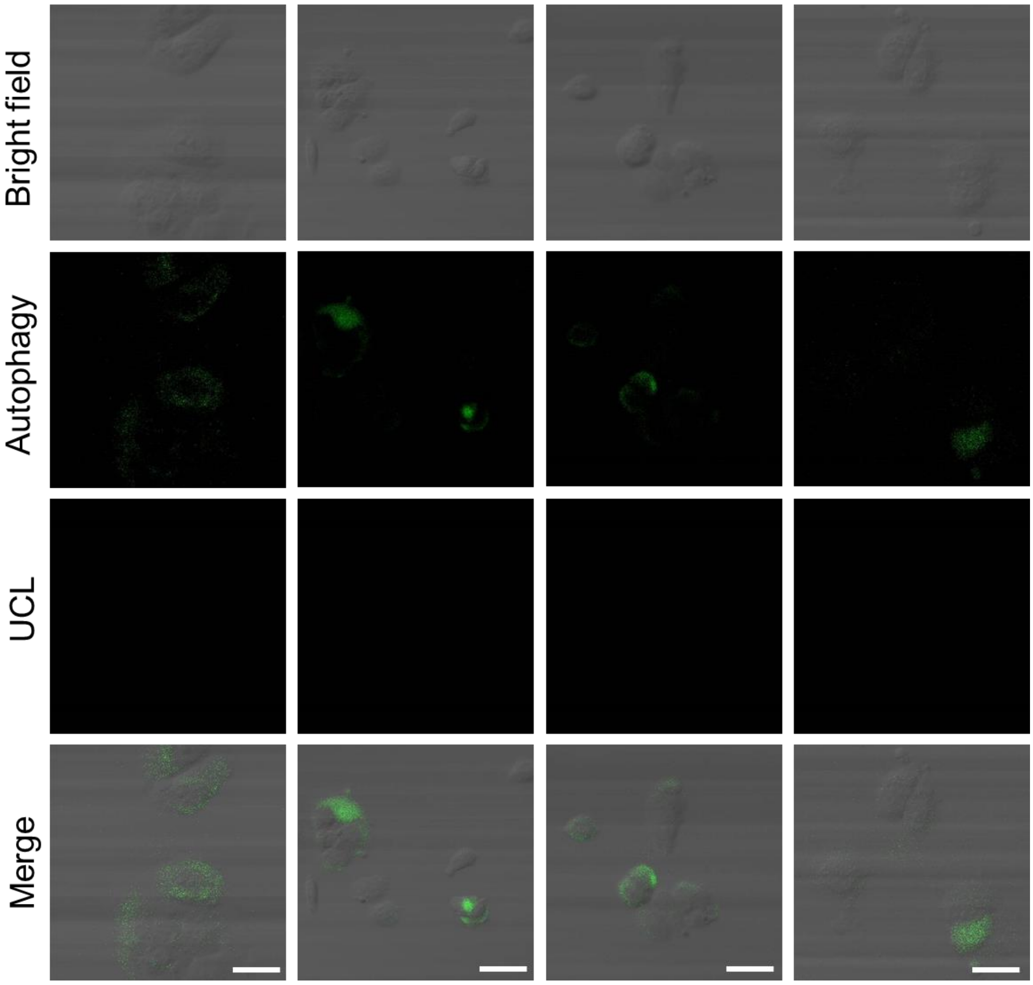

Supplementary Figure 36. The confocal images of MCF-7 cells (5×10^7) treated by D-GSH-modified gold nanoparticle (40 nM) for 12h. Scale bar 20 μm. All experiments were performed in triplicate.

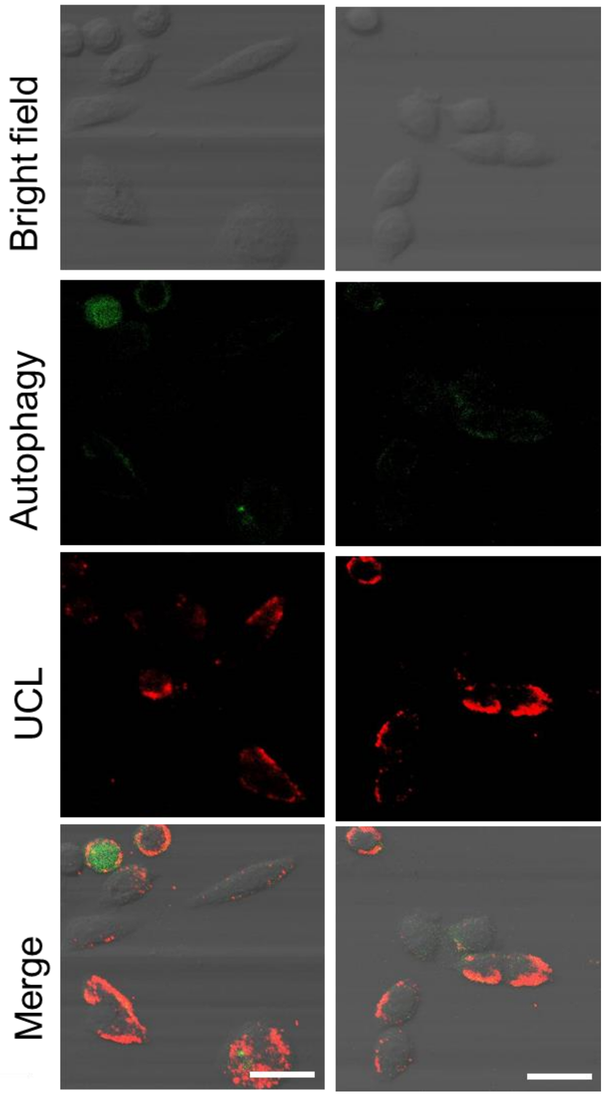

Supplementary Figure 37. The confocal images of MCF-7 cells (5×10^7) treated by D-GSH-modified UCNP (40 nM) for 12h. Scale bar 20 µm. All experiments were performed in triplicate.

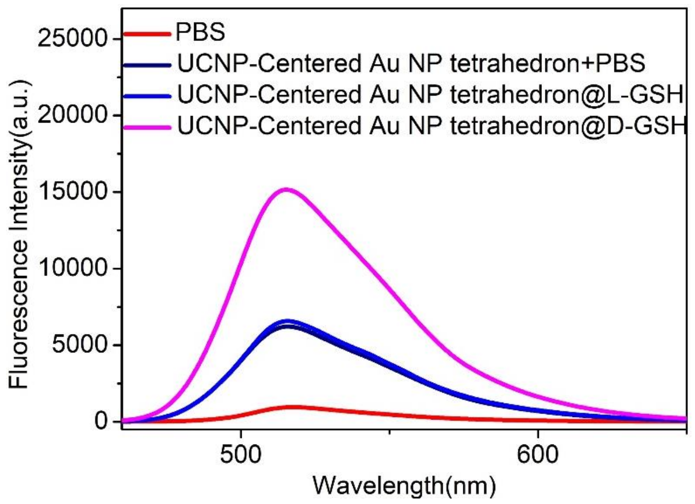

Supplementary Figure 38. The ROS production of MCF-7 cells (5×10^7) treated by PBS, UCNP-Centered Au NP tetrahedron (40 nM) in PBS, L-GSH modified UCNP-Centered Au NP tetrahedron (40 nM), and D-GSH modified UCNP-Centered Au NP tetrahedron (40 nM) for 12h measured by H2DCFDA (Invitrogen™) (GSH concentration is 5 μM). All experiments were performed in triplicate.
